# A Preclinical Systematic Review of the Effects of Chronic Exercise on Autophagy-Related Proteins in Aging Skeletal Muscle

**DOI:** 10.3389/fphys.2022.930185

**Published:** 2022-07-14

**Authors:** Cenyi Wang, Jiling Liang, Yuanyuan Ren, Jielun Huang, Baoming Jin, Guodong Wang, Ning Chen

**Affiliations:** ^1^ School of Physical Education and Sports Science, Soochow University, Suzhou, China; ^2^ Tianjiu Research and Development Center for Exercise Nutrition and Foods, Hubei Key Laboratory of Exercise Training and Monitoring, College of Health Science, Wuhan Sports University, Wuhan, China

**Keywords:** chronic exercise, autophagy, aging, skeletal muscle, systematic review

## Abstract

**Background:** Exercise is one of the most effective interventions for preventing and treating skeletal muscle aging. Exercise-induced autophagy is widely acknowledged to regulate skeletal muscle mass and delay skeletal muscle aging. However, the mechanisms underlying of the effect of different exercises on autophagy in aging skeletal muscle remain unclear.

**Methods:** A systematic review was performed following an electronic search of SCOPUS, PubMed, Web of Science, ScienceDirect, and Google Scholar and two Chinese electronic databases, CNKI and Wan Fang. All articles published in English and Chinese between January 2010 and January 2022 that quantified autophagy-related proteins in aging skeletal muscle models.

**Results:** The primary outcome was autophagy assessment, indicated by changes in the levels of any autophagy-associated proteins. A total of fifteen studies were included in the final review. Chronic exercise modes mainly comprise aerobic exercise and resistance exercise, and the intervention types include treadmill training, voluntary wheel running, and ladder training. LC3, Atg5-Atg7/9/12, mTOR, Beclin1, Bcl-2, p62, PGC-1α, and other protein levels were quantified, and the results showed that long-term aerobic exercise and resistance exercise could increase the expression of autophagy-related proteins in aging skeletal muscle (*p* < 0.05). However, there was no significant difference in short term or high-intensity chronic exercise, and different types and intensities of exercise yielded different levels of significance for autophagy-related protein expression.

**Conclusion:** Existing evidence reveals that high-intensity exercise may induce excessive autophagy, while low-intensity exercise for a short period (Intervention duration <12 weeks, frequency <3 times/week) may not reach the threshold for exercise-induced autophagy. Precise control of the exercise dose is essential in the long term to maximize the benefits of exercise. Further investigation is warranted to explore the relationship between chronic exercise and different exercise duration and types to substantiate the delaying of skeletal muscle aging by exercise.

## Introduction

Skeletal muscle is one of the most dynamic tissues in the human body. Skeletal muscle can adapt to many metabolic and physiological requirements imposed by various stressors as a highly plastic tissue. The high adaptability of skeletal muscle is necessary for the body to regulate cellular homeostasis in response to various stress intensities and durations (Beyfuss and Hood, 2018). Skeletal muscle is an essential dynamic organ in the human body, accounting for about 40% of the total body weight, which contains 50%–75% of the whole-body protein, and accounts for about 30%–50% of the total protein turnover. In general, the skeletal muscle mass depends on the balance between protein synthesis and degradation, which are highly sensitive to physical activity/exercise, nutritional status, injury or disease (Frontera and Ochala, 2015).

With the progressive aging of society, the prevalence of comorbidities and senile diseases, especially sarcopenia, characterized by the decline in skeletal muscle mass and strength, have also increased (Distefano and Goodpaster, 2018). The pathological mechanism of sarcopenia is mirrored by a significant reduction of skeletal muscle fibers and muscle volume and a significant decrease in skeletal muscle mass or strength (Tarantino et al., 2015). Sarcopenia usually stems from the imbalance between muscle protein synthesis and degradation caused by aging and is accompanied by a series of dysregulations in signal pathways. Its essence can be summarized as the excessive net protein degradation resulting in loss of skeletal muscle mass since protein degradation is more remarkable than synthesis (Dhillon and Hasni, 2017). Autophagy plays a vital role in controlling skeletal muscle quality, and its means of degradation is mainly ubiquitin-proteasome and autophagy-lysosome degradation pathways. Although the specific mechanism is unclear at present, excessive autophagy can lead to muscle atrophy; as a result, activating autophagy in muscles to promote mitochondrial productivity is extremely necessary for improving exercise function.

Exercise is a newly defined effective stimulus that can induce the activation of autophagy. Over the years, much emphasis has been placed on autophagy regulation in muscle cells by exercise. It has been found that exercise could activate skeletal muscle autophagy by increasing the LC3-I/LC3-II ratio (Grumati et al., 2011). Exercise is currently one of the most effective intervention approaches to prevent and treat skeletal muscle aging. Appropriate and personalized exercise can enhance the body adaptability and function of elderly individuals, improve skeletal muscle function and quality of life, and effectively increase the health level to prolong life (Cruz-Jentoft et al., 2010). In recent years, intervention studies have been carried out worldwide on delaying skeletal muscle aging, comparing effectiveness between different exercise modes and different exercise intensities, including resistance exercise and aerobic exercise, and significant differences were observed. However, it should be noted that for the elderly population, high-intensity exercise usually brings a certain degree of risk; and due to the age-related decline in skeletal muscle mass and strength and the occurrence of osteoporosis, a short period of moderate to low-intensity resistance exercise or aerobic exercise may not be enough to cause the energy “burning” of muscle cells in the aging body, resulting in insignificant effects of weight control or muscle mass maintenance (Moreira et al., 2017). Thus, chronic exercise represents an effective rehabilitation approach to improve exercise adaptability and delay skeletal muscle tissue aging by encouraging exercising at a moderate intensity and reasonable load in young and middle-aged adults as a long-term lifestyle habit.

Although previous studies have reviewed the related mechanisms of exercise on skeletal muscle autophagy, and many experimental studies have assessed the effect of exercise on autophagy activity in skeletal muscle, the research objects and conclusions were inconsistent (Grumati et al., 2011; Kim et al., 2012; Moreira et al., 2017; Kwon et al., 2018). Indeed, significant heterogeneity surrounded the exercise intervention methods, post-exercise sampling time, studied tissues, the nutritional level of experimental subjects before exercise, exercise intervention cycles, etc. Therefore, this article aims to review the latest available evidence, taking the effect of chronic exercise on the autophagy activation of aging skeletal muscle as the research object obtain a standardized framework to analyze and evaluate present related research. Importantly, this review provides novel insights into the association between chronic exercise and the level of autophagy in aging skeletal muscle.

## Methods

### Search Strategy

We reported the systematic review following the Preferred Reporting Items for Systematic Reviews and Meta-Analyses (PRISMA) checklist guidelines. This systematic review examined the role of chronic exercise on autophagy in aging skeletal muscles. Articles published in English and Chinese language were retrieved from five English electronic databases (SCOPUS, PubMed, Web of Science, ScienceDirect, and Google Scholar) and two Chinese electronic databases (China National Knowledge Infrastructure, Wan Fang) using the key terms “exercise,” “chronic exercise,” “physical activity,” “aging,” “skeletal muscle,” “autophagy,” “protein,” and “gene.” We also conducted a manual search in the Soochow University and Wuhan Sports University libraries to prevent retrieval bias. The searches of the databases were completed between September 2021 and January 2022. Only full articles in English or Chinese were included in the assessment. The preliminary study for comparison only involved aging animals and cell culture models. Significant studies published in this field involving older human subjects were discussed where applicable. To maintain the focus of this systematic review, they were not compiled for analysis in the data tables. All literature data published between January 2010 and January 2022 were included in the review.

### Eligibility Criteria

Databases were systematically searched for studies, and their eligibility for inclusion was determined. The research was initially filtered based on whether the title contained any keywords. Subsequently, the abstract was read to further screen keywords and appropriate eligibility criteria (Table 1). The full text was read and then analyzed. When an article met the eligibility criteria, it was included in the systematic review. Duplicated articles and unpublished research were excluded at this stage.

**TABLE 1:** Inclusion and exclusion criteria. At each stage of the research analysis for inclusion review, papers were included and excluded according to the above criteria.

Stage	Stage description	Inclusion criteria	Exclusion criteria
Ⅰ	Journal title analysis	Keywords: exercise, chronic exercise, physical activity, aging, skeletal muscle, autophagy, protein, gene	① Keywords: human, younger age, review, myocardial, brain, liver cells, tissues (other than skeletal muscle); ② Languages other than English or Chinese; ③ Human model; ④ Aging animals and cell culture models (other than skeletal muscle); ⑤Review or methods paper; ⑥Abstract or full-test is unavailable; ⑦ Not original study; ⑧ Missing data; ⑨ Proteins or genes outside the range.
Ⅱ	Abstract analysis	①Skeletal muscle tissue; ②Animal or cell model; ③Autophagy signaling activation; ④Chronic exercise.	
Ⅲ	Full text analysis	①Skeletal muscle tissue; ②Animal or cell model; ③Autophagy signaling activation; ④Chronic exercise; ⑤Proteins include any one of the following: LC3, AMPK, Atg5/7/9/12, IGF-1, mTOR, FoxO3, Beclin1, Bcl-2, p62, PGC-1α.	

Note: LC3, microtubule-associated protein-1 lightchain 3; AMPK, adenosine monophosphate-activated protein kinase; Atg, autophagy-related; mTOR, mammalian target of rapamycin; IGF-1, Insulin-like Growth Factors 1; Bcl-2, B-cell lymphoma 2; PGC-1α, peroxisome proliferator-activated receptor γ coactivator1-α.

### Data Extraction

The studies were organized based on the mechanism of autophagy activation, and chronic exercise intervention was divided into two groups according to the duration and type of intervention: long-term vs. short-term exercise and resistance vs. aerobic exercise. The included studies fulfilled at least one of the two categories. Data extraction was used to evaluate the study methodology of the effect of exercise on the autophagy level in skeletal muscle cells, as well as the application and relevance of the model. Two reviewers (CY and YY) created, undertook, and crosschecked a data extraction standard form to record the data including the first author, publication year, research model, location of skeletal muscles, sample size, gender, exercise Intervention (frequency, intensity, duration), outcome measures and outcome data. Any disagreements between two reviewers were resolved by discussion; otherwise, a third reviewer (Ning) would be consulted. Whenever necessary, the corresponding author was contacted to request additional information or for clarification where discrepancies existed. Qualitative analysis was performed to compare the effectiveness of models based on different types of chronic exercise activating autophagy-related proteins to regulate the level of autophagy in aging-induced skeletal muscle atrophy. No meta-analysis was conducted using this data.

### Quality Assessment of Included Studies

Two authors (CY and JL) independently assessed methodological quality and risk of bias assessment of the included studies using the Collaborative Approach to Meta Analysis and Review of Animal Experimental Studies (CAMARADES) 10-item quality checklist (Macleod et al., 2004). Each study was given a quality score out of 10 points. Two authors (CY and JL) independently assessed the study quality. Any discrepancy was resolved by discussion, and the corresponding author (GD) adjudicated whenever the disagreement could not be resolved.

## Results

### Study Inclusion

**FIGURE 1:**
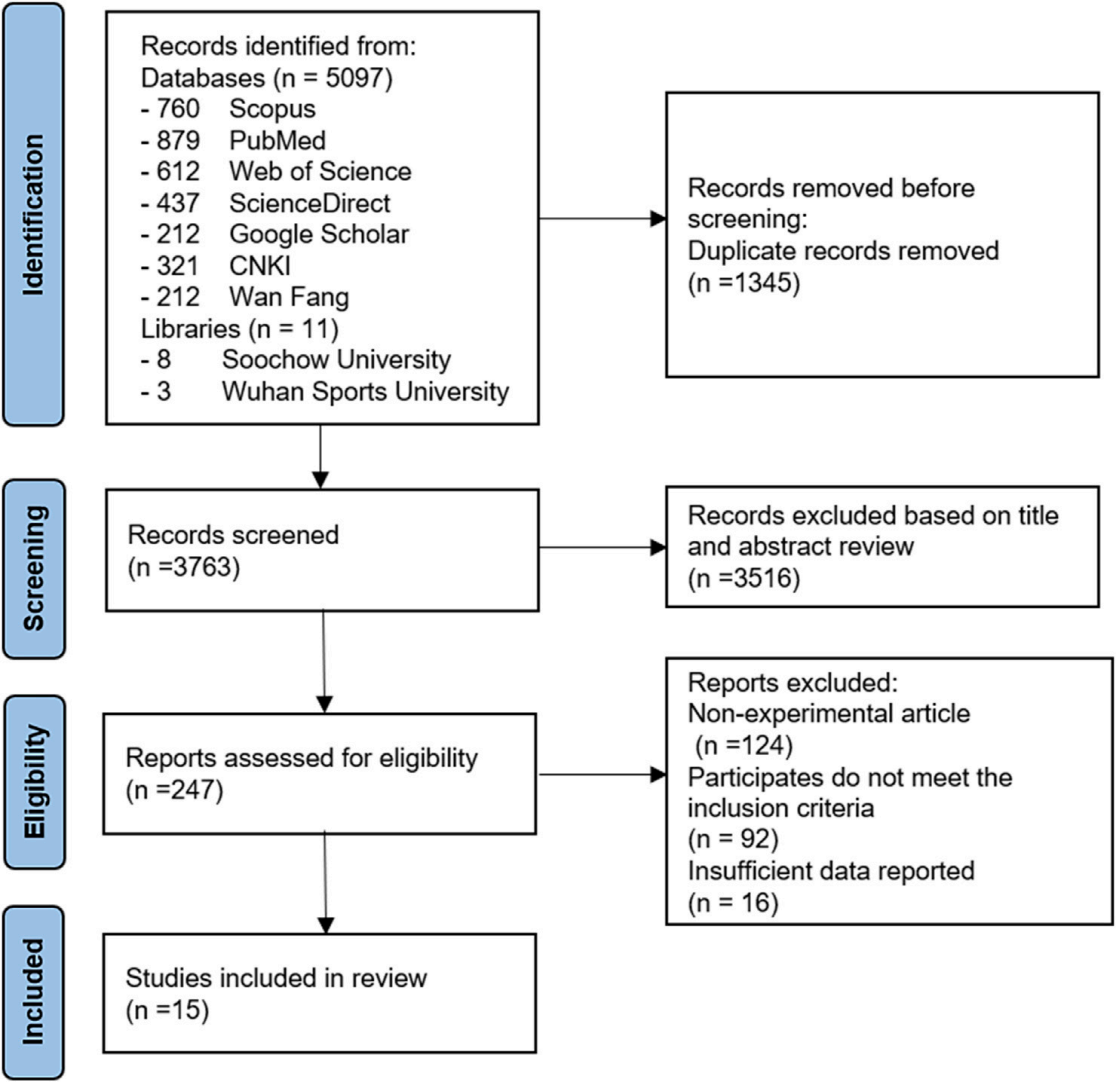
Flow diagram of study selection process.

The Medical Subject Heading terms used for the initial search included “chronic exercise,” “exercise,” “physical activity,” “aging,” “skeletal muscle,” “autophagy,” and their combinations. A total of 5,108 trial articles were identified and evaluated. After excluding duplicates, eliminating studies based on the title, and reading the full text, fifteen articles met the criteria for inclusion and were eventually included in the review. The selected studies were from academic journals. The flowchart details of the literature included in the study are shown in Figure 1.

### General Characteristics of the Included Studies

A total of 382 skeletal muscle samples were included in the full text. Only one study (White et al., 2016) included on both males and females, while the remaining studies only males (*n* = 12) or females (*n* = 1) (Dethlefsen et al., 2018). Seven studies involved rats (*n* = 3 for Sprague Dawley rats, *n* = 3 for Wistar rats and *n* = 1 for Fischer 344 rats), and eight studies used mice (*n* = 4 for C57BL/6 mice, *n* = 2 for ICR mice, *n* = 1 for C57BL/6 whole-body PGC-1α knock out and wild-type mice, and *n* = 1 for *VCP* ^R155H/+^mice). Regarding the selection of skeletal muscle samples, most studies selected the gastrocnemius and quadriceps muscles, and only four articles selected the plantaris, soleus, and extensor digitorum muscles. In nine studies, elderly subjects were selected for long-term exercise; in six studies, young subjects were selected for lifelong exercise. In terms of the form of exercise intervention, all studies chose aerobic/resistance exercise, and one study (Zeng et al., 2020) added an alternating exercise (aerobic + resistance exercise) intervention group and an aerobic/resistance exercise intervention group. The specific methods of exercise intervention included treadmill exercise, voluntary wheel running, and ladder exercise with load. The intervention lasted from 6 to 85 weeks, and the intervention frequency was three or five times per week (Table 2).

**TABLE 2:** A summary of the reviewed articles.

Study (author, year)	Research model	Location of skeletal muscles	Sample size	Age at start (months)	Exercise intervention				Outcome measures	Main findings of exercise in the intervention group
					Duration	Frequency	Intensity	Mode		
Wohlgemuth et al. (2010)	Male Fischer 344 rats	Plantaris	EG:20 CG:19	3	84–85 weeks	Not reported	1,145 ± 248 m/day	Lifelong VWR	Beclin-1, Atg7, Atg9, LC3-II/I ratio	No significant effects for Beclin-1; ↑Atg7 (*p* < 0.05); ↑Atg9 (p =0.022); LC3-II/I ratio was 90% higher in CG and EG (*p* = 0.2).
Lin et al. (2013)	Male Sprague Dawley rats	Gastrocnemius	EG:30 CG:8	18	8 weeks	3 d/week	0%, 30%, 50%, 70% maximum weight RE separately; speed:15 m/min, slope:35°	RE	Bcl-2	↑Bcl-2 (p <0.01 for 30% EG, p < 0.05 for 50% EG); No significant effects for Bcl-2 in 0% EG and 70% EG.
Luo et al. (2013)	Male Sprague Dawley rats	Gastrocnemius	EG:19 CG:20	18-20	9 weeks	3 d/week	10 RM	RE	Beclin-1, Atg5-12, Atg7, LC3-II/ I ratio, p62, AMPK, IGF1, FoxO3a	↑Beclin-1, Atg7, Atg5/12, AMPK, IGF1, FOXO3a (*p* < 0.01); ↓LC3-II/ I ratio, p62 (*p* < 0.01).
Nalbandian et al. (2013)	Male VCPR155H/+mice	Quadriceps	EG:8–10 CG:8–10	18	6 weeks	3 d/week	10–30 min (progressive)	TE	LC3-II/ I ratio, p62	↓LC3-II/ I ratio, p62 (*p* < 0.01).
Zhao et al. (2013)	Male C57BL/6 mice	Gastrocnemius	EG:15(TE=8, RE=7) CG:6	3	68 weeks	3 d/week	TE:30 min/d; RE:2 Sets/d	Lifelong TE; RE	LC3-II/I ratio, Beclin1	↑LC3-II/I ratio (TE and RE, *p* < 0.05); ↑Beclin1 (TR, *p* < 0.05).
Lee et al. (2015)	Male C57BL/6 mice	Soleus	EG:20(LE=6, HE=7) CG:10	24	2 weeks	5 d/week	50 min/d, LE:8.8 m/min; HE:17.45 m/min	TE	Beclin-1, LC3-II/ I ratio, p62, PGC-1α	No significant effects for Beclin-1 and LC3-II/I ratio; ↑PGC-1α (2.2-fold higher in LE, 1.1-fold in HE, *p* < 0.01); ↓p62 (reduced by 35% in the LE, *p* < 0.05, but no changes in HE vs. CG).
Ziaaldini et al. (2015)	Male wistar rats	Quadriceps	EG:6 CG:6	8	6 weeks	Not reported	30-60 min/d, 60%VO2max	TE	mTOR, Bcl-2	No significant effects for mTOR, Bcl-2.
White et al. (2016)	Male andfemale C57BL/6J mice	Quadriceps	EG:13(M=6, F=7) CG:19(M=10, F=9)	15	34 weeks	4,5 d/week	30 min/d	VWR (resistance)	LC3-II/ I ratio; p62	No significant effects for p62; ↑LC3-II/ I ratio (elevated by 48% in M and 62% in F, *p* < 0.01).
Zhao et al. (2016)	Male C57BL/6 mice	Gastrocnemius	EG:18(TE=9, RE=9) CG:9	17	16 weeks	3 d/week	TE:40 min/d; RE:2 Sets /d	TE; RE	LC3-II/ I ratio, Beclin-1	↓Beclin1(TE and RE, *p* < 0.05); ↓LC3-II/ I ratio (TE, *p* < 0.05); ↑LC3-II/ I ratio (RE, *p* < 0.05).
Ribeiro et al. (2017)	Male Wistar rats	Gastrocnemius + Soleus	EG:6 CG:6	20	12 weeks	3 d/week	Progressive load of 65%–95% maximumcarrying capacity	RE	PGC-1α, IGF1, mTOR	↑PGC-1α, IGF1, mTOR (*p* < 0.05).
Bahreinipour et al. (2018)	Male Sprague Dawley rats	Soleus + Extensor digitorum longus	EG:8 CG:8	23–24	10 weeks	5 d/week	15-50 min (progressive), LE	TE	PGC-1α	↑PGC-1α (*p* < 0.05).
Dethlefsen et al. (2018)	Female C57BL/6 PGC-1α KO and WT mice (intercross breeding)	Quadriceps	EG:5-8 CG:5-8	3	48 weeks	Not reported	Not reported	Lifelong running wheel	LC3-II/ I ratio, LC3I, LC3II, Bcl-2, PGC-1α, p62	No significant effects for LC3I, LC3II, Bcl-2, p62; ↑PGC-1α (*p* < 0.05); ↓LC3-II/ I ratio (50% lower than CG, *p* < 0.05).
Zeng et al. (2020)	Male wistar rats	Gastrocnemius	EG:40 CG:10	21	12 weeks	TE, RE, alternating ex (TE +RE): 3d/week; VWR: Free	TE: 4.2–12 m/min, 60 min(progressive); RE: 3 times/set, 2 sets /d; alternating ex (TE +RE): TE/RE protocols; VWR: Free	TE; RE; alternating ex (TE+ RE); VWR	Beclin-1, LC3-II/ I ratio, p62, mTOR, FOXO3a, Bcl-2, AMPK, PGC-1α	↑Beclin-1(*p* < 0.01 for all EGs except TE p <0.05); ↑LC3-II/ I ratio, Bcl-2, AMPK (*p* < 0.01); ↑PGC-1α (*p* < 0.01 for RE and VWR, *p* < 0.05 for TE and alternating ex); ↓p62, p-mTOR/mTOR (*p* < 0.01); ↓pFOXO3a/FOXO3a(*p* < 0.01 for TE and RE, *p* < 0.05 for alternating ex and VWR).
Liang et al. (2021)	Male ICR mice	Gastrocnemius	EG:10 CG:10	3	56 weeks	5 d/week	60 min	Lifelong TE	Beclin-1, LC3-II/LC3-I ratio, p62, mTOR, Bcl-2, AMPK, PGC-1α	↑Beclin-1, LC3-II/ I ratio, AMPK (*p* < 0.001); ↑Bcl-2(*p* < 0.01); ↑PGC-1α (*p* < 0.05); ↓p62, p-mTOR/mTOR(*p* < 0.001);
Zheng and Liang (2021)	Male ICR mice	Gastrocnemius	EG:6 CG:6	3	57 weeks	3 d/week	3 times/set, 2 Sets/d	Lifelong RE	Beclin-1, Bcl-2	↑Beclin-1, Bcl-2(*p* < 0.001)

Note: KO, knock out; WT, wild type; M, male; F, female; LE. low intensity exercise; HE, high intensity exercise; VWR, voluntary wheel running; RE, resistance exercise training; TE, treadmill exercise; Ex, exercise; RM, repetition maximum.

### Study Quality

The CAMARADES 10-item quality checklist was used to determine the quality and risk of bias of each study. The quality of the included studies ranged from 3 to 7 points, with an average score of 4.93. All studies were published in a peer-reviewed journal. Two studies did not report control of temperature (Dethlefsen et al., 2018; Zheng and Liang, 2021). Three studies did not describe random allocation to treatment or control (Nalbandian et al., 2013; Ziaaldini et al., 2015; White et al., 2016), and only one study mentioned blinded induction of the model (Liang et al., 2021). Eight studies used anesthetics without significant intrinsic neuroprotective activity (Wohlgemuth et al., 2010; Lin et al., 2013; Luo et al., 2013; Ziaaldini et al., 2015; White et al., 2016; Ribeiro et al., 2017; Bahreinipour et al., 2018; Dethlefsen et al., 2018). An appropriate animal model (aged, diabetic, or hypertensive) was described in five studies (Lin et al., 2013; Zhao et al., 2016; Zeng et al., 2020; Liang et al., 2021; Zheng and Liang, 2021). No study described a sample size calculation or blinded assessment of outcome. Compliance with animal welfare regulations was not described in five studies (Zhao et al., 2013; Lee et al., 2015; Ziaaldini et al., 2015; Zhao et al., 2016; Dethlefsen et al., 2018), and potential conflicts of interest were not mentioned in five studies (Luo et al., 2013; Zhao et al., 2013; Zhao et al., 2016; Lin et al., 2020; Zheng and Liang, 2021). Detailed results of methodological quality are shown in Table 3.

**TABLE 3:** Quality assessment of the included studies.

Study (author, year)	(1)	(2)	(3)	(4)	(5)	(6)	(7)	(8)	(9)	(10)	Score
Wohlgemuth et al. (2010)	✓	✓	✓			✓			✓	✓	6
Lin et al. (2013)	✓	✓	✓			✓	✓		✓		6
Luo et al. (2013)	✓	✓	✓			✓			✓		5
Nalbandian et al. (2013)	✓	✓				Nk			✓	✓	4
Zhao et al. (2013)	✓	✓	✓			Nk					3
Lee et al. (2015)	✓	✓	✓			Nk				✓	4
Ziaaldini et al. (2015)	✓	✓				✓				✓	4
White et al. (2016)	✓	✓				✓			✓	✓	5
Zhao et al. (2016)	✓	✓	✓			Nk	✓				4
Ribeiro et al. (2017)	✓	✓	✓			✓			✓	✓	6
Bahreinipour et al. (2018)	✓	✓	✓			✓			✓	✓	6
Dethlefsen et al. (2018)	✓		✓			✓				✓	4
Zeng et al. (2020)	✓	✓	✓			Nk	✓		✓	✓	6
Liang et al. (2021)	✓	✓	✓	✓	✓	Nk	✓		✓	✓	8
Zheng and Liang (2021)	✓		✓			Nk	✓		✓		4

Note: studies fulfilling the criteria of the following: (1), peer reviewed publication; (2), control of temperature; (3), random allocation to treatment or control; (4), blinded induction of model; (5), blinded assessment of outcome; (6), use of anesthetic without significant intrinsic neuroprotective activity; (7), appropriate animal model (aged, diabetic, or hypertensive); (8), sample size calculation; (9), compliance with animal welfare regulations; and (10), statement of potential conflict of interests. Nk, not known.

### Effectiveness

The studies in this systematic review analyzed multiple autophagy-related protein markers LC3, Atg5/7/9/12, mTOR, Beclin1, Bcl-2, p62, PGC-1α, FoxO3, IGF-1, AMPK, and other proteins (Table 2). LC3-II/LC3-I ratio was reported in ten studies, of which four (Luo et al., 2013; Nalbandian et al., 2013; Zhao et al., 2016; Dethlefsen et al., 2018) indicated that chronic exercise reduced the LC3-II/LC3-I ratio (*p* < 0.05), while six (Wohlgemuth et al., 2010; Zhao et al., 2013; White et al., 2016; Zhao et al., 2016; Zeng et al., 2020; Liang et al., 2021) showed that chronic exercise would increase the LC3-II/LC3-I ratio of aging skeletal muscle (*p* < 0.05). One study (Lee et al., 2015) found no significant change in the ratio of LC3-II/LC3-I. Two studies (Wohlgemuth et al., 2010; Luo et al., 2013) showed that long-term aerobic and resistance exercise could increase the Atg7, Atg9, and Atg5/12 protein levels in skeletal muscle (*p* < 0.05).

Eight studies (Wohlgemuth et al., 2010; Luo et al., 2013; Zhao et al., 2013; Lee et al., 2015; Zhao et al., 2016; Zeng et al., 2020; Liang et al., 2021; Zheng and Liang, 2021) reported Beclin1 protein expression in skeletal muscle; five studies showed that chronic exercise increased the expression of Beclin1, and one indicated a decrease in expression (*p* < 0.05); there was no significant difference in Beclin1 level between the intervention and control groups in the other two studies. Five studies (Lin et al., 2013; Ziaaldini et al., 2015; Dethlefsen et al., 2018; Liang et al., 2021; Zheng and Liang, 2021) reported Bcl-2 protein markers, of which three reported a significant increase in Bcl-2 protein expression, while the remaining two showed no significant difference. It is worth noting that Lin et al. (2013) divided long-term resistance exercises into four groups: 0%, 30%, 50%, and 70%. The results indicated that the expression of Bcl-2 protein in skeletal muscle increased with 30% and 50% resistance exercise, while no significant difference was found in the 0% and 75% intervention groups. Six studies (Lee et al., 2015; Ribeiro et al., 2017; Bahreinipour et al., 2018; Dethlefsen et al., 2018; Zeng et al., 2020; Liang et al., 2021) found that chronic exercise could significantly increase the PGC-1α protein levels in skeletal muscle (*p* < 0.05).

A total of seven pieces of literature (Luo et al., 2013; Nalbandian et al., 2013; Lee et al., 2015; White et al., 2016; Dethlefsen et al., 2018; Zeng et al., 2020; Liang et al., 2021) reported the p62 protein marker. Five studies showed that the expression of p62 protein decreased (*p* < 0.01), and two studies showed no significant difference. Three articles (Luo et al., 2013; Zeng et al., 2020; Liang et al., 2021) reported the AMPK protein in skeletal muscle, and two reported the IGF-1 protein level, demonstrating a significant increase in related proteins (*p* < 0.05). As for mTOR and FoxO3 gene outcomes, mTOR expression levels were documented in four articles (Ziaaldini et al., 2015; Ribeiro et al., 2017; Zeng et al., 2020; Liang et al., 2021), and FoxO3 results in two articles (Luo et al., 2013; Zeng et al., 2020). Zeng et al. (2020) found that 12-week exercise significantly reduced the expression of the ratio of p-mTOR/mTOR and p-FoxO3/FoxO3(*p* < 0.01), while 9-week and 12-week long-term resistance exercise significantly increased FoxO3 and mTOR gene expression in skeletal muscle(*p* <0.05). Besides increases in autophagy-related protein markers, some studies assessed muscle mass, muscle/body mass ratio, running distance, and speed, and the results showed that chronic exercise intervention positively impacts these markers.

## Discussion

**FIGURE 2:**
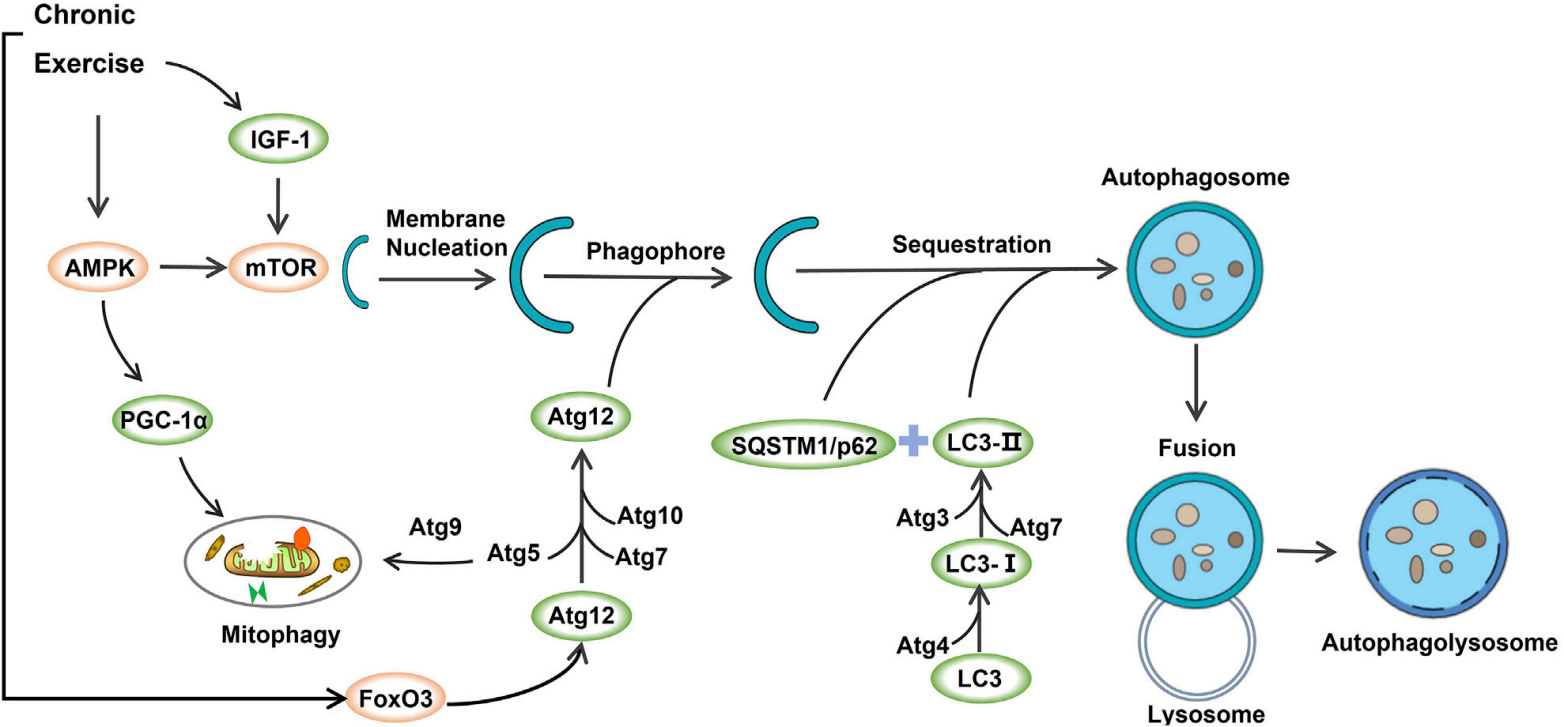
The underlying mechanisms of exercise-induced autophagy in aging-induced skeletal muscle atrophy.

This study systematically reviewed the effects of chronic exercise-induced autophagy on skeletal muscle to delay aging-induced skeletal muscle atrophy, and summarized several key findings (see Figure 2).

### The Role of AMPK, IGF-1, PGC-1α, and mTOR in Mediating Autophagy Induced by Chronic Exercise

The present research investigated the influence of long-term exercise on skeletal muscle autophagy and assessed adaptive changes in basal autophagy and autophagy flux after exercise intervention. mTOR and AMPK have been established as essential indicators for detecting skeletal muscle nutrition and energy efficiency during exercise response (Pantovic et al., 2017). As a highly conserved serine/threonine-protein kinase, mTOR can combine with other ligands to form mTORC1 and participate in the inhibition of autophagy (Wullschleger et al., 2006). In addition to activating the catabolic process in cell organisms and inhibiting the biosynthesis of lipids, proteins, and carbohydrates, AMPK is reportedly a critical factor in the initiation of autophagy (Hong et al., 2005). In this review, four articles analyzed mTOR, and three articles examined the role of AMPK protein in regulating autophagy and delaying muscle atrophy. The included studies suggested that aerobic exercise for 60 min 3–5 times a week for more than 12 weeks could significantly increase the expression of mTOR and AMPK and thereby induce autophagy. Reduced AMPK activity associated with senescence may lead to decreased mitochondrial function and dysregulated intracellular lipid metabolism. Interestingly, the activation of AMPK and inhibition of mTOR in muscle after acute exercise have been documented in previous animal and human studies, while long-term aerobic exercise can regulate the phosphorylation of mTOR by increasing AMPK activity; meanwhile, AMPK can inhibit mTOR signaling through tuberous sclerosis complex 1/2 (TSC1/2)/Ras homolog enrichment in brain (Rheb) pathway, thereby inducing autophagy in skeletal muscle, attenuating cellular senescence and apoptosis signals, thus reducing muscle loss (Nobukini and Thomas, 2004).

It is widely acknowledged that IGF-1 plays a key role in cell proliferation and differentiation during muscle regeneration, and high IGF-1 levels can inhibit muscle atrophy during aging (Schiaffino and Mammucari, 2011). Moreover,IGF-1 depends on the mTOR signaling pathway and promotes mitochondrial biogenesis in skeletal muscle cells (Hu et al., 2021). The studies included in this review reported that long-term resistance exercise 3 times per week over 9 weeks could increase the expression of IGF-1 in aging cells, thereby delaying the aging process of skeletal muscle. It has been shown that the transcriptional co-activator PGC-1α (one of the regulatory genes of mitochondrial biogenesis) is also regulated by various upstream factors, including AMPK and p38, which can promote the expression of PGC-1α (Vainshtein et al., 2015). Moreover, AMPK can promote PGC-1α transcription while activating PGC-1α, and p38 promotes the phosphorylation of PGC-1α. An increasing body of evidence suggests that PGC-1α expression can be significantly increased by long-term aerobic or resistance exercise, or alternating aerobic and resistance exercise. By analyzing the molecular mechanism, chronic exercise could improve mitochondrial quality control in aging skeletal muscle by activating PGC-1α through phosphorylation of AMPK and p38 (Irrcher et al., 2008). However, it is worth mentioning that low-intensity aerobic exercise (2-week treadmill exercise) in 24-month mice was remarkedly effective in increasing the expression of PGC-1α compared to high intensity exercise (Lee et al., 2015), suggesting that strenuous exercise can inhibit the up-regulation of mitochondrial function in aging skeletal muscle to a certain extent, and low-intensity exercise might be more advantageous in delaying aging-induced skeletal muscle atrophy.

### The Role of Beclin1 and Bcl-2 in Mediating Autophagy Induced by Chronic Exercise

Beclin1, a homolog of the yeast autophagy gene Atg6, is a crucial factor involved in the formation and maturation of autophagosomes and enhancing autophagy activity. However, this signaling pathway is also regulated by the expression of Bcl-2 (Russell et al., 2013). Under normal physiological conditions, Bcl-2 in cells has a strong binding ability to Beclin1 to maintain basic levels of autophagy. During the aging process of skeletal muscle, the expression of Beclin1/Bcl-2 complex decreases to initiate apoptosis, whereas long-term regular aerobic or resistance exercise can enhance the expression of Beclin1/Bcl-2 complex and delay the decrease of skeletal muscle mass. It should be noted that the difference in sampling time after exercise intervention can also affect the expression of Beclin1 and Bcl-2, influencing the changes in autophagy activity of skeletal muscle cells. Interestingly, it has been reported that the expression of Beclin1-Bcl-2 complex immediately decreased 15 min after exercise intervention, while it was almost undetected 30 min later (Akimoto et al., 2005). Four studies included in this review indicated no significant changes in Beclin1 and Bcl-2 compared with the control group after chronic exercise intervention; one article found that after 8 weeks of 30% and 50% weight-loading exercise, Bcl-2 protein expression increased, while there was no significant change in 0 % and 75% weight-loading intervention groups. Therefore, taking into account the exercise intensity of chronic exercise and the timeliness of cellular Beclin1 and Bcl-2 proteins after exercise, the sampling time should be emphasized when assessing changes in skeletal muscle autophagy after chronic exercise in the future.

### The Role of LC3 and p62 in Mediating Autophagy Induced by Chronic Exercise

p62 is a multifunctional scaffold protein composed of multiple domains. It has been shown that autophagy adaptors p62 and LC3 can interact with ubiquitinated protein by autophagy to achieve the specific recognition, separation, and transportation of p62, thereby releasing nutrients into the cell cycle of skeletal muscle (Rogov et al., 2014). Hence, p62 interacts with LC3 to wrap the ubiquitinated substrate, exhibiting decreased expression during autophagy. The animal homolog of Atg8 protein is LC3. During autophagy, LC3-I is continuously lipidated to form LC3-II to promote the formation of an autophagic membrane; thus, the ratio of LC3-II/LC3-I is generally used to evaluate the functional level of autophagy (He et al., 2015). In this review, ten studies included LC3-II/LC3-I ratio outcomes, and seven included P62 protein outcomes. Overwhelming evidence substantiates that long-term resistance or aerobic exercise can down-regulate the expression of p62 and increase the ratio of LC3-II/LC3-I, thereby improving autophagy and stabilizing the quality of mitochondria. Interestingly, a study reported that the expression of P62 protein was similar to that of PGC-1α, and the expression of p62 protein was reduced by 35% after 2 weeks of low-intensity treadmill exercise, while there was no significant difference in the expression of p62 in high-intensity treadmill exercise compared with the non-exercise control group (Lee et al., 2015). This finding further supports our hypothesis that excessive high-intensity exercise may inhibit autophagy in aging skeletal muscle. Additionally, four included studies showed that the ratio of LC3-II/LC3-I decreased after aerobic/resistance exercise, indicating that chronic exercise inhibits autophagy initiation in aging skeletal muscle cells. Nevertheless, previous studies have demonstrated that LC3 and Beclin-1 levels were comparable in rats after treadmill exercise of 60 min per day for 5 days (Smuder et al., 2011), suggesting that short-term aerobic exercise does not induce adaptive changes in autophagy. In this review, among the related studies that showed a decreased LC3-II/LC3-I ratio, the exercise intervention time was predominantly 10–40 min for 6–16 weeks, 3 times a week. The related literature reported an increased LC3-II/LC3-I ratio after exercise for 60 min 5 times per week for than 12 weeks, consistent with our findings. Indeed, it should be borne in mind that chronic exercise-induced autophagy in aging skeletal muscle cells may require the exercise cycle to reach a certain threshold.

### The Role of Atg5-Atg9, Atg7, Atg12, and FoxO3a in Mediating Autophagy Induced by Chronic Exercise

It is well-established that a series of complexes composed of products encoded by the Atg gene can participate in the formation of autophagosomes in skeletal muscle cells. The complete membrane protein Atg9 is the key to autophagosome formation, and Atg5/Atg12 directly affects the extension and maturation of autophagosomes. In contrast, Atg7 is an essential autophagosome marker that controls the extension of the autophagosome membrane and plays a crucial role in forming autophagosomes (Fujitani et al., 2009; Fogel et al., 2013; Crisol et al., 2018; Lin et al., 2020). Two studies showed that chronic exercise could increase the expression of autophagy-related proteins Atg5, Atg9, Atg7, and Atg12, thus inducing autophagy in plantaris and gastrocnemius cells.

FoxO genes are widely expressed in various eukaryotes and are related to the regulation of autophagy. It has been shown that FoxO3a can induce autophagy by regulating the transcription of autophagy-related genes such as LC3, Atg5, and Atg12 (Jamart et al., 2012; Liu et al., 2019). Additionally, the post-translational modification of FoxO3a plays a critical role in regulating the autophagy flux in skeletal muscle. AMPK can promote the phosphorylation of FoxO3a at Ser588/Ser413, enhance transcriptional activity, and promote autophagy (Krawiec et al., 2007). Accordingly, FoxO3a-induced skeletal muscle autophagy is a vital pathway independent of other signaling pathways. Importantly, this study showed that long-term resistance/aerobic exercise and alternating aerobic and resistance exercises could affect FoxO3a gene expression and promote autophagy in aging skeletal muscle cells. Indeed, different exercise modes and types may have heterogeneous effects on the expression of FoxO3a. Interestingly, ladder climbing or treadmill running alone may have a more significant response to the autophagy induced by FoxO3a than voluntary wheel running and ladder climbing combined with treadmill running.

## Limitations

Several limitations were present in this preclinical systematic review. First, the study designs of the few included studies may not be as reliable as others. Given that two studies did not specify whether the intervention group was randomly assigned, the effect of selection bias on our study findings could not be ruled out. Moreover, since the frequency and intensity of exercise intervention in the experimental group in three studies were not mentioned, we could not conduct a comprehensive comparative analysis of relevant study protocol and reported outcomes with other studies. It is worth mentioning that the initiation of exercise interventions varied in the included studies, ranging from 3 to 24 months of age, with chronic exercise intervention lasting from 2 weeks to life-time intervention. The significant difference in the age of initiation and duration of the intervention may alter the effect size of the relevant outcome, thereby affecting the analysis results.

Furthermore, a wide range of training modes was analyzed in this study, including aerobic exercise and resistance exercise, and the exercise types involve treadmill exercise, ladder climbing exercise, voluntary wheel running, etc. Significant differences in the number, frequency and intensity of the exercise program in intervention groups were observed, even for ladder climbing training. Any variation of these parameters may result in different autophagy responses. Moreover, differences between aging animal models included in the study and the location of selected skeletal muscle fiber samples are crucial factors to be considered. The differences in life span, behavioral characteristics, and motor function between C57BL/6 mice and SD rats, Wistar rats and ICR mice may also lead to different autophagy responses after chronic exercise. Moreover, the sampling points of skeletal muscles included quadriceps, gastrocnemius, soleus, etc. We acknowledge that the effects of different types of exercises on skeletal muscle, warranting further research on stress and adaptation mechanisms induced by chronic exercise against autophagy.

## Conclusion

To the best of our knowledge, this is the first systematic review to summarize the effects of chronic exercise on autophagy-related proteins in aging skeletal muscle and analyzed the regulation of chronic exercise on autophagy-related proteins in different forms, types, and intensities, as well as various signaling pathways involved in maintaining the homeostasis of aging skeletal muscle cells. The results showed that long-term aerobic and resistance exercise could regulate autophagy-related proteins to induce skeletal muscle autophagy and delay the loss of muscle mass, while different exercise modes such as the age of exercise initiation, duration, and intensity of exercise may have heterogeneous effects on the expression of autophagy-related proteins. High-intensity exercise may induce excessive autophagy, while low-intensity and short-term exercise intervention (Duration: less than 12 weeks, frequency: less than 3 times per week) may not reach the threshold for exercise-induced autophagy. Accordingly, in order to achieve the optimal exercise benefit, it is necessary to pay attention to scientific control of chronic exercise dose. In summary, research on the influence of chronic exercise on the autophagy of aging skeletal muscle cells is still in its preliminary stages. It is essential to comprehensively explore the activation mechanism of chronic exercise on autophagy and the adaptive changes of autophagy under different intervention durations and types of chronic exercise to provide the foothold for exploring the effects of chronic exercise on delaying aging skeletal muscle and promoting physical health.

## Data Availability

The original contributions presented in the study are included in the article/supplementary material, further inquiries can be directed to the corresponding authors.

## References

[B1] Akimoto T. Pohnert S. C. Li P. Zhang M. Gumbs C. Rosenberg P. B. (2005). Exercise Stimulates Pgc-1α Transcription in Skeletal Muscle through Activation of the P38 MAPK Pathway. J. Biol. Chem. 280, 19587–19593. 10.1074/jbc.m408862200 15767263

[B2] Bahreinipour M.-A. Joukar S. Hovanloo F. Najafipour H. Naderi V. Rajiamirhasani A. (2018). Mild Aerobic Training with Blood Flow Restriction Increases the Hypertrophy Index and MuSK in Both Slow and Fast Muscles of Old Rats: Role of PGC-1α. Life Sci. 202, 103–109. 10.1016/j.lfs.2018.03.051 29604268

[B3] Beyfuss K. Hood D. A. (2018). A Systematic Review of P53 Regulation of Oxidative Stress in Skeletal Muscle. Redox Rep. 23, 100–117. 10.1080/13510002.2017.1416773 29298131 PMC6748683

[B4] Crisol B. M. Lenhare L. Gaspar R. S. Gaspar R. C. Muñoz V. R. Da Silva A. S. R. (2018). The Role of Physical Exercise on Sestrin1 and 2 Accumulations in the Skeletal Muscle of Mice. Life Sci. 194, 98–103. 10.1016/j.lfs.2017.12.023 29273527

[B5] Cruz-Jentoft A. J. Baeyens J. P. Bauer J. M. Boirie Y. Cederholm T. Landi F. (2010). Sarcopenia: European Consensus on Definition and Diagnosis: Report of the European Working Group on Sarcopenia in Older People. Age Ageing 39, 412–423. 10.1093/ageing/afq034 20392703 PMC2886201

[B6] Dethlefsen M. M. Halling J. F. Møller H. D. Plomgaard P. Regenberg B. Ringholm S. (2018). Regulation of Apoptosis and Autophagy in Mouse and Human Skeletal Muscle with Aging and Lifelong Exercise Training. Exp. Gerontol. 111, 141–153. 10.1016/j.exger.2018.07.011 30030137

[B7] Dhillon R. J. S. Hasni S. (2017). Pathogenesis and Management of Sarcopenia. Clin. Geriatric Med. 33, 17–26. 10.1016/j.cger.2016.08.002 PMC5127276 27886695

[B8] Distefano G. Goodpaster B. H. (2018). Effects of Exercise and Aging on Skeletal Muscle. Cold Spring Harb. Perspect. Med. 8, a029785. 10.1101/cshperspect.a029785 28432116 PMC5830901

[B9] Fogel A. I. Dlouhy B. J. Wang C. Ryu S.-W. Neutzner A. Hasson S. A. (2013). Role of Membrane Association and Atg14-dependent Phosphorylation in Beclin-1-Mediated Autophagy. Mol. Cell. Biol. 33, 3675–3688. 10.1128/mcb.00079-13 23878393 PMC3753860

[B10] Frontera W. R. Ochala J. (2015). Skeletal Muscle: a Brief Review of Structure and Function. Calcif. Tissue Int. 96, 183–195. 10.1007/s00223-014-9915-y 25294644

[B11] Fujitani Y. Ebato C. Uchida T. Kawamori R. Watada H. (2009). β-Cell Autophagy: A Novel Mechanism Regulating β-cell Function and Mass- Lessons from β-cell-specific Atg7-Deficient Mice. Islets 1, 151–153. 10.4161/isl.1.2.9057 21099263

[B12] Grumati P. Coletto L. Schiavinato A. Castagnaro S. Bertaggia E. Sandri M. (2011). Physical Exercise Stimulates Autophagy in Normal Skeletal Muscles but Is Detrimental for Collagen VI-deficient Muscles. Autophagy 7, 1415–1423. 10.4161/auto.7.12.17877 22024752 PMC3288016

[B13] He R. Peng J. Yuan P. Xu F. Wei W. (2015). Divergent Roles of BECN1 in LC3 Lipidation and Autophagosomal Function. Autophagy 11, 740–747. 10.1080/15548627.2015.1034404 25955014 PMC4509441

[B14] Hong S.-P. Momcilovic M. Carlson M. (2005). Function of Mammalian LKB1 and Ca2+/Calmodulin-dependent Protein Kinase Kinase α as Snf1-Activating Kinases in Yeast. J. Biol. Chem. 280, 21804–21809. 10.1074/jbc.m501887200 15831494

[B15] Hu B. Li H. Zhang X. (2021). A Balanced Act: The Effects of GH-GHR-IGF1 Axis on Mitochondrial Function. Front. Cell. Dev. Biol. 9, 630248. 10.3389/fcell.2021.630248 33816476 PMC8012549

[B16] Irrcher I. Ljubicic V. Kirwan A. F. Hood D. A. (2008). AMP-Activated Protein Kinase-Regulated Activation of the PGC-1α Promoter in Skeletal Muscle Cells. PLoS One 3, e3614. 10.1371/journal.pone.0003614 18974883 PMC2570798

[B17] Jamart C. Francaux M. Millet G. Y. Deldicque L. Frère D. Féasson L. (2012). Modulation of Autophagy and Ubiquitin-Proteasome Pathways during Ultra-endurance Running. J. Appl. Physiology 112, 1529–1537. 10.1152/japplphysiol.00952.2011 22345427

[B18] Kim Y. A. Kim Y. S. Song W. (2012). Autophagic Response to a Single Bout of Moderate Exercise in Murine Skeletal Muscle. J. Physiol. Biochem. 68, 229–235. 10.1007/s13105-011-0135-x 22205581

[B19] Krawiec B. J. Nystrom G. J. Frost R. A. Jefferson L. S. Lang C. H. (2007). AMP-Activated Protein Kinase Agonists Increase mRNA Content of the Muscle-specific Ubiquitin Ligases MAFbx and MuRF1 in C2C12 Cells. Am. J. Physiology-Endocrinology Metabolism 292, E1555–E1567. 10.1152/ajpendo.00622.2006 17264220

[B20] Kwon I. Jang Y. Cho J.-Y. Jang Y. C. Lee Y. (2018). Long-term Resistance Exercise-Induced Muscular Hypertrophy Is Associated with Autophagy Modulation in Rats. J. Physiol. Sci. 68, 269–280. 10.1007/s12576-017-0531-2 28213823 PMC10718009

[B21] Lee S. Kim M. Lim W. Kim T. Kang C. (2015). Strenuous Exercise Induces Mitochondrial Damage in Skeletal Muscle of Old Mice. Biochem. Biophysical Res. Commun. 461, 354–360. 10.1016/j.bbrc.2015.04.038 25887799

[B22] Liang J. Zhang H. Zeng Z. Wu L. Zhang Y. Guo Y. (2021). Lifelong Aerobic Exercise Alleviates Sarcopenia by Activating Autophagy and Inhibiting Protein Degradation via the AMPK/PGC-1α Signaling Pathway. Metabolites 11, 323. 10.3390/metabo11050323 34069829 PMC8157243

[B23] Lin T.-Y. Chan H.-H. Chen S.-H. Sarvagalla S. Chen P.-S. Coumar M. S. (2020). BIRC5/Survivin Is a Novel ATG12-ATG5 Conjugate Interactor and an Autophagy-Induced DNA Damage Suppressor in Human Cancer and Mouse Embryonic Fibroblast Cells. Autophagy 16, 1296–1313. 10.1080/15548627.2019.1671643 31612776 PMC7469615

[B24] Lin W. Wang X. Wen X. Xu G. Huang L. (2013). Effects of Resistance Training on the Aging Rats Gastrocnemius of Apoptosis Regulatory Genes and Mitochondrial Membrane Potential. J. Tianjin Univ. Sport 28, 419–421. 10.13297/j.cnki.issn1005-0000.2013.05.011

[B25] Liu Y. Li J. Shang Y. Guo Y. Li Z. (2019). CARM1 Contributes to Skeletal Muscle Wasting by Mediating FoxO3 Activity and Promoting Myofiber Autophagy. Exp. Cell. Res. 374, 198–209. 10.1016/j.yexcr.2018.11.024 30500392

[B26] Luo L. Lu A.-M. Wang Y. Hong A. Chen Y. Hu J. (2013). Chronic Resistance Training Activates Autophagy and Reduces Apoptosis of Muscle Cells by Modulating IGF-1 and its Receptors, Akt/mTOR and Akt/FOXO3a Signaling in Aged Rats. Exp. Gerontol. 48, 427–436. 10.1016/j.exger.2013.02.009 23419688

[B27] Macleod M. R. O’Collins T. Howells D. W. Donnan G. A. (2004). Pooling of Animal Experimental Data Reveals Influence of Study Design and Publication Bias. Stroke 35, 1203–1208. 10.1161/01.str.0000125719.25853.20 15060322

[B28] Moreira O. C. Estébanez B. Martínez-Florez S. De Paz J. A. Cuevas M. J. González-Gallego J. (2017). Mitochondrial Function and Mitophagy in the Elderly: Effects of Exercise. Oxid. Med. Cell. Longev. 2017, 2012798. 10.1155/2017/2012798 28900532 PMC5576425

[B29] Nalbandian A. Nguyen C. Katheria V. Llewellyn K. J. Badadani M. Caiozzo V. (2013). Exercise Training Reverses Skeletal Muscle Atrophy in an Experimental Model of VCP Disease. PLoS One 8, e76187. 10.1371/journal.pone.0076187 24130765 PMC3794032

[B30] Nobukini T. Thomas G. (2004). The mTOR/S6K Signalling Pathway: the Role of the TSC1/2 Tumour Suppressor Complex and the Proto-Oncogene Rheb. Novartis Found. Symp. 262, 148–154. 10.1002/0470869976.ch9 15562827

[B31] Pantovic A. Bosnjak M. Arsikin K. Kosic M. Mandic M. Ristic B. (2017). *In Vitro* antiglioma Action of Indomethacin Is Mediated via AMP-Activated Protein kinase/mTOR Complex 1 Signalling Pathway. Int. J. Biochem. Cell. Biol. 83, 84–96. 10.1016/j.biocel.2016.12.007 27988363

[B32] Ribeiro M. B. T. Guzzoni V. Hord J. M. Lopes G. N. Marqueti R. d. C. De Andrade R. V. (2017). Resistance Training Regulates Gene Expression of Molecules Associated with Intramyocellular Lipids, Glucose Signaling and Fiber Size in Old Rats. Sci. Rep. 7, 8593. 10.1038/s41598-017-09343-6 28819168 PMC5561018

[B33] Rogov V. Dötsch V. Johansen T. Kirkin V. (2014). Interactions between Autophagy Receptors and Ubiquitin-like Proteins Form the Molecular Basis for Selective Autophagy. Mol. Cell. 53, 167–178. 10.1016/j.molcel.2013.12.014 24462201

[B34] Russell R. C. Tian Y. Yuan H. Park H. W. Chang Y.-Y. Kim J. (2013). ULK1 Induces Autophagy by Phosphorylating Beclin-1 and Activating VPS34 Lipid Kinase. Nat. Cell. Biol. 15, 741–750. 10.1038/ncb2757 23685627 PMC3885611

[B35] Schiaffino S. Mammucari C. (2011). Regulation of Skeletal Muscle Growth by the IGF1-Akt/PKB Pathway: Insights from Genetic Models. Skelet. Muscle, 1, 4. 10.1186/2044-5040-1-4 21798082 PMC3143906

[B36] Smuder A. J. Kavazis A. N. Min K. Powers S. K. (2011). Exercise Protects against Doxorubicin-Induced Markers of Autophagy Signaling in Skeletal Muscle. J. Appl. Physiology 111, 1190–1198. 10.1152/japplphysiol.00429.2011 21778418

[B37] Tarantino U. Piccirilli E. Fantini M. Baldi J. Gasbarra E. Bei R. (2015). Sarcopenia and Fragility Fractures: Molecular and Clinical Evidence of the Bone-Muscle Interaction. J. Bone Jt. Surg. 97, 429–437. 10.2106/jbjs.n.00648 25740034

[B38] Vainshtein A. Tryon L. D. Pauly M. Hood D. A. (2015). Role of PGC-1α during Acute Exercise-Induced Autophagy and Mitophagy in Skeletal Muscle. Am. J. Physiology-Cell Physiology 308, C710–C719. 10.1152/ajpcell.00380.2014 PMC4420796 25673772

[B39] White Z. Terrill J. White R. B. Mcmahon C. Sheard P. Grounds M. D. (2016). Voluntary Resistance Wheel Exercise from Mid-life Prevents Sarcopenia and Increases Markers of Mitochondrial Function and Autophagy in Muscles of Old Male and Female C57BL/6J Mice. Skelet. Muscle 6, 45. 10.1186/s13395-016-0117-3 27964759 PMC5155391

[B40] Wohlgemuth S. E. Seo A. Y. Marzetti E. Lees H. A. Leeuwenburgh C. (2010). Skeletal Muscle Autophagy and Apoptosis during Aging: Effects of Calorie Restriction and Life-Long Exercise. Exp. Gerontol. 45, 138–148. 10.1016/j.exger.2009.11.002 19903516 PMC2829942

[B41] Wullschleger S. Loewith R. Hall M. N. (2006). TOR Signaling in Growth and Metabolism. Cell. 124, 471–484. 10.1016/j.cell.2006.01.016 16469695

[B42] Zeng Z. Liang J. Wu L. Zhang H. Lv J. Chen N. (2020). Exercise-Induced Autophagy Suppresses Sarcopenia through Akt/mTOR and Akt/FoxO3a Signal Pathways and AMPK-Mediated Mitochondrial Quality Control. Front. Physiol. 11, 583478. 10.3389/fphys.2020.583478 33224037 PMC7667253

[B43] Zhao Y. Dai Y. Chen C. He Y. Lu J. (2016). The Alteration of Autophagy-Related Gene in Gastrocnemius Muscle of Mice with Sarcopenia after Endurance Exercise and Resistance Exercise. Chin. J. Sports Med. 35, 449–455. 10.16038/j.1000-6710.2016.05.007

[B44] Zhao Y. Dai Y. Lu J. Chen C. Guo Y. (2013). Effects of Different Exercises Type on Sarcopenia-Associated ATGS Expression in Skeletal Muscle of Natural Aging Mouse. J. Beijing Sport Univ. 36, 82–87. 10.19582/j.cnki.11-3785/g8.2013.12.016

[B45] Zheng J. Liang J. (2021). Mechanism of Long-Term Resistance Exercise Inhibiting Skeletal Muscle Degradation in Aging Mice. J. Southwest China Normal Univ. Nat. Sci. Ed. 46, 95–100. 10.13718/j.cnki.xsxb.2021.08.016

[B46] Ziaaldini M. M. Koltai E. Csende Z. Goto A. W. Boldogh I. Taylor A. W. (2015). Exercise Training Increases Anabolic and Attenuates Catabolic and Apoptotic Processes in Aged Skeletal Muscle of Male Rats. Exp. Gerontol. 67, 9–14. 10.1016/j.exger.2015.04.008 25910622

